# The antimicrobial susceptibility of *Stenotrophomonas maltophilia *isolates using three different methods and their genetic relatedness

**DOI:** 10.1186/1471-2180-5-24

**Published:** 2005-05-09

**Authors:** Müşerref Tatman-Otkun, Şaban Gürcan, Burçin Özer, Bayram Aydoslu, Şebnem Bukavaz

**Affiliations:** 1 Medical Faculty, Department of Microbiology and Clinical Microbiology, Trakya University, Turkey; 2 Institute of Health Science, Trakya University, Turkey; 3 Medical Faculty, Department of Clinical Bacteriology and Infectious Diseases, 22030- Edirne, TURKEY Trakya University, Turkey

## Abstract

**Background:**

*Stenotrophomonas maltophilia *is inherently resistant to many antimicrobials. So far, antimicrobial susceptibility tests for *S. maltophilia *have not been fully standardized. The purpose of the study was to compare the susceptibility of *S. maltophilia *isolates against seven different antimicrobials using three different methods and to investigate their genetic relatedness.

**Results:**

Although trimethoprim/sulfamethoxazole (SXT) and ciprofloxacin have the lowest MIC values, SXT (98.1%) and ticarcillin/clavulanate (TLc) (73.1%) were found to be the most effective antimicrobials by agar dilution method, which was in accordance with the breakpoints established by NCCLS. Disc diffusion and E-test was in agreement with agar dilution method for SXT. When the isolation dates, clinics, antibiotyping, and AP-PCR data were investigated, two small outbreaks consisting of five and three cases were determined.

**Conclusion:**

By using the NCCLS criteria, disc diffusion and E-test were unreliable alternative methods for *S. maltophilia*, except for SXT. However, the significance of these data should be confirmed by further experimental and clinical studies.

## Background

Infections with *Stenotrophomonas maltophilia *are increasingly encountered especially in debilitated or immune suppressed patients [1,2]. Nosocomial infections of the respiratory, urinary, central nervous, musculoskeletal, skin-soft tissue systems and of the gastrointestinal tract, and bacteremia, endocarditis and eye infections occur in sensitive individuals [2-4].

*S. maltophilia *is inherently resistant to many antimicrobials. Additional resistance develops against cephalosporins and aminoglycosides because of decreased outer membrane permeability and via at least two types of beta-lactamases [5]. Recently, resistance to floroquinolones via efflux system has been reported [6]. Also, antimicrobial susceptibility tests for *S. maltophilia *have not been so far fully standardized [2]. These problems raise difficulties for the choice of antimicrobials in *S. maltophilia *infections.

In this study, disc diffusion and E-test methods were compared with the agar dilution method in *S. maltophilia *strains against seven different antimicrobials. We also investigated the genetic relatedness of *S. maltophilia *isolates by the arbitrarily primed PCR (AP-PCR).

## Results

*S. maltophilia *strains were mostly isolated from the lower respiratory tract (n = 20), and from urine (n = 11) and blood (n = 7) samples, and the majority were from the clinics of Paediatrics (n = 14), Neurology (n = 9), Nephrology (n = 8), Chest Diseases (n = 4) and Intensive Care Units (n = 4). Only six strains were isolated in 1998; however the isolation rate steadily increased throughout the years and reached 17 strains in 2002. In particular, the annual isolation rate in the clinics of Paediatrics and Neurology was only one or two until 2001, however it increased to six in 2001 and five in 2002 in Paediatrics. Also, six strains were isolated in Neurology in 2002.

Although trimethoprim/sulfamethoxazole (SXT) and ciprofloxacin (CIP) have the lowest minimal inhibitory concentration (MIC) values, the most effective antimicrobials were SXT and ticarcillin/clavulanate (TLc), which was in accordance with the breakpoints established by The National Committee for Clinical Laboratory Standards (NCCLS). The rate of susceptibilities obtained by the disc diffusion and E-test methods were similar to the agar dilution test; however, false susceptibility for CIP by both tests (p < 0.001) and for TLc by the E-test were obtained (p < 0.04) (Table 1). The disc diffusion and E-test methods showed a good agreement with agar dilution method for SXT (Table 2). Rates of correlations were poor for the other antimicrobials.

**Figure 1:**
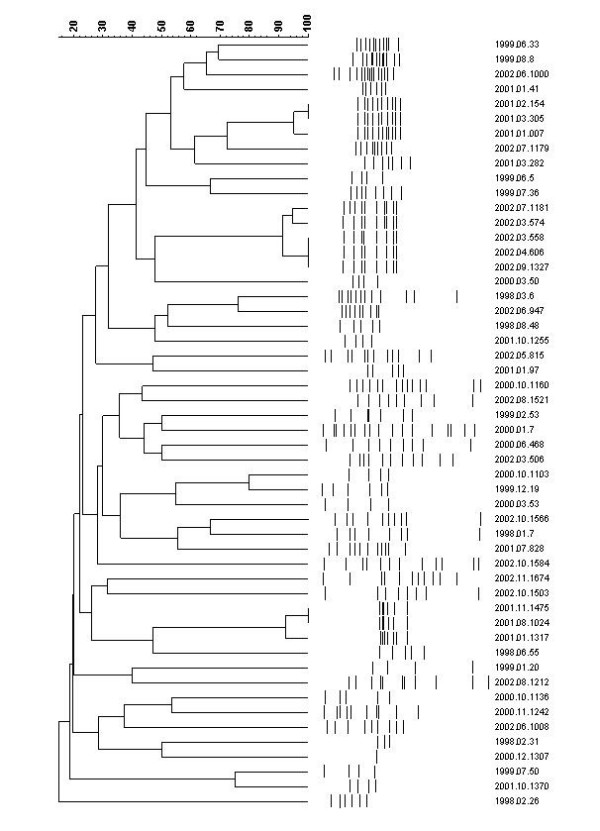
AP-PCR profiles of *S. maltophilia *strins including statistical analysis and dendrogram showing the genetic relationship between strains.

A total of 44 different patterns of 52 strains were obtained by The arbitrarily primed PCR (AP-PCR). Three small clusters were observed. All of five strains in pattern l were isolated in the Neurology clinic between March and September 2002. All three strains in pattern ll were isolated in the Paediatrics clinic between January and March 2001, and three strains in pattern lll were isolated in the Paediatrics, Nephrology and Chest Diseases clinics between January and November 2001. The antimicrobial susceptibility results supported the AP-PCR method for patterns l and ll, but not for pattern III. The strains in pattern I were resistant to ceftazidime (CAZ), cefepime (CPM), piperacillin (PIP) and piperacillin/tazobactam (PTZ), and susceptible to TLc, CIP, and SXT. The strains in pattern II were susceptible to all antimicrobial except for CIP. The dendrogram showed a Dice similarity coefficient ranging from14.8 to 100% (Figure 1).

## Discussion

*S. maltophilia *has low pathogenicity, but it has emerged as an important nosocomial pathogen. Patients infected with *S. maltophilia *usually have underlying immunodeficiency or history of long-term or multiple hospitalizations, exposure to invasive devices and/or broad spectrum antimicrobials [2]. This organism, most frequently, causes lower respiratory and urinary tract infections and may result in secondary bacteremia [2]. Our results supported these observations.

According to the recommendations of the NCCLS [7,8], agar dilution method should be used in order to detect antimicrobial susceptibility of *S. maltophilia *strains. Since the dilution methods are more cumbersome or expensive than the disc diffusion or E-test methods in routine clinical microbiology laboratories, the aim of this study was to compare the performance of these latter methods with agar dilution method.

The NCCLS had not defined the criteria for disc diffusion method for *S. maltophilia *by the year 2004. So, breakpoints for other bacteria from the NCCLS comments have been tried to be adapted in various studies [6,14]. The best correlated results have been obtained with those recommended for *Pseudomonas aeruginosa *[6]. In 2004, the NCCLS recommended disc diffusion breakpoints for minocycline, levofloxacin, and SXT. Nevertheless, we tested other antimicrobials frequently used in nosocomial infections and interpreted these antimicrobial susceptibilities using the NCCLS criteria established for *P. aeruginosa*.

Our results showed that the most effective antimicrobials against *S. maltophilia *were SXT and TLc, as observed by several authors [6,14]. Nevertheless, the resistance rates for other antimicrobials in our study were extremely high. Resistance to beta-lactams in *S. maltophilia *is primarily intrinsic and mediated by inducible beta-lactamases (L1 and L2) that hydrolyses virtually all classes of beta-lactams [2]. Although many authors have tested piperacillin (with or without tazobactam) and cefepime against *S. maltophilia*, they are not suitable for the treatment of *S. maltophilia *infections. L2 beta-lactamase is susceptible to clavulanic acid, so TLc is preferred to PTZ [2,15]. However, Carrol et al. [1] determined that there was an obvious increase in the level of resistance to TLc when they prolonged the susceptibility tests up to 48 hours. This finding makes the in vitro efficiency of TLc disputable. The NCCLS has recently recommended 20–24 hours incubation for *S. maltophilia*, and so we did not evaluate the tests after 48 hours incubation [9].

The correlations between in vitro susceptibility methods for *S. maltophilia *show variations [10,14]. While Nicodemo et al. [10] stated that the disc diffusion tests have an excellent correlation with agar dilution for several antimicrobials; Pankuch et al. [14] used the breakpoint values for Enterobacteriaceae recommended by the NCCLS and found a high level of discordance for PTZ, TLc, CIP. Also, in our study, poor agreement was observed in the alternative test methods except for SXT. In a study where the correlation of E-test with agar dilution method for 16 antimicrobials in 176 clinical isolates were investigated, the authors found an excellent correlation and recommended E-test as an alternative susceptibility test [16]. Major and very major errors were very low in this latter study and were similar to our results for all the antimicrobials except for CIP and CAZ by both the disc diffusion and by the E-test. However most of the discordant susceptibility rates among the three methods evaluated were due to high occurrence of minor errors in our study. The MIC values of our strains cumulated close to breakpoints. For instance, the susceptibility breakpoint of CIP established by the NCCLS is 1 mg/L [7] and equals to MIC_50 _value of our strains. Moreover MIC values for CIP were 1 mg/L and 2 mg/L of 18 and 17 strains, respectively. The same applies for CAZ and CPM also. Therefore, minor variations caused to change in the susceptibility categories from intermediate susceptibility to susceptibility or resistance or vice versa. If variations in ± 1 doubling dilutions between different methods were to be accepted as essential agreement suggested by Pankuch et al [14] then our error rates would have been much more smaller.

Tracking of *S. maltophilia *isolates has a great importance to reveal their outbreaks, to determine the distribution routes and to take preventive measures. However, biotyping and antibiotyping methods are not reliable due to the relative metabolic inactivity and multiresistance of these isolates. More recently, genotypic methods have been developed and used with success to discriminate for phenotypically indistinguishable bacteria. AP-PCR is one of the most preferred molecular typing methods for this aim, because the results can be obtained rapidly even in a clinical laboratory. Also, it can be applied to a wide range of bacterial species by using almost the same materials and equipment [2,13]. We determined two small outbreaks consisting of five cases in the Neurology and three cases in the Paediatrics clinics by using AP-PCR method. Isolation dates, clinics, and antibiotyping data have also supported these results. On the other hand, the third cluster (strains from pattern III) suggests that the same strain can persist for a long time in hospital. The first strain from pattern III were isolated from the Pediatrics clinic in January 2001. The second and third strains were isolated from the Nephrology clinic in August 2001 and from the Chest Diseases clinic in November 2001, respectively. Reported nosocomial outbreaks due to *S. maltophilia *are generally short termed. [2]. There is no report of prolonged transmission extending up to 11 months for *S. maltophilia*. Valdezate et al [17] concluded that the epidemiological relationship among different *S. maltophilia *isolates needed to be analysed because unexpected results could be obtained. Antibiotic susceptibility profiles also supported that isolates in pattern III were independent isolates having same genotypes.

## Conclusion

In conclusion, the disc diffusion and the E-test methods were unreliable alternative methods for *S. maltophilia*, except for SXT. However, significance of these data should be confirmed by further experimental and clinical studies.

## Methods

### Bacteria

The 52 *S. maltophilia *strains (one per patient) that were isolated from nosocomial infections between 1998 and 2002 in the Hospital of Trakya University were included in the study. The strains were identified by conventional bacteriological methods and were stored at -70°C in skim-milk media (Becton Dickinson, USA). Before the study, they were twice passaged onto 5% sheep blood agar and the identification was confirmed by Crystal ID Enteric-nonfermenter (Becton Dickson, USA).

### Antimicrobial susceptibility tests

The drug powders for the agar dilution test were obtained from the following suppliers: Ceftazidime pentahydrate (Glaxo-Welcome, UK), CPM (Bristol-Myers Squibb, USA), PIP and tazobactam (Lederle, USA), ticarcillin disodium and clavulanate lithium (GlaxoSmithKline, UK), CIP (Bayer, Turkey), trimethoprim and sulfamethoxazole (Roche, Turkey). Standard antimicrobial discs (Oxoid, UK) were used for the disc diffusion tests and E-test strips were supplied by AB Biodisk, Sweden.

Antimicrobial susceptibility tests were carried out using the disc diffusion and agar dilution techniques as described by NCCLS [7,8]. The Agar dilution and E-test results were interpreted using the NCCLS criteria established for non-enterobacteriaceae, and the disc diffusion test was interpreted using the criteria established for *P. aeruginosa *[7-9]. The E-test technique was carried out according to the manifacturer's instructions. The tests were evaluated after 20–24 hours incubation at 35°C and were repeated if they were found to be discordant.

*Escherichia coli *the American Type Culture Collection (ATCC) 25922 and *P. aeruginosa *ATCC 27853 were used as quality control strains.

### Definitions

The agar dilution method was accepted as the reference method. Categorical agreement was defined if the tests results were within the same susceptibility category, and errors of disc diffusion and E-test methods were determined as follows: Very major error; (resistant by reference method, susceptible by test method); major error; (susceptible by reference method, resistant by test method); and minor error; (intermediate result was obtained by one method but not the other) [10]. Percentage errors were calculated based on the total number of isolates which were tested. A good agreement was defined as complete category agreement over 90% and the total of very major and major errors below 5% [11].

### Arbitrarily primed PCR

The method of vanCouwenberghe et al. [12] was used for the preparation of the DNA and AP-PCR, with minor modifications. Briefly, after an overnight culture at 37°C in 5% sheep blood agar, the bacteria were suspended in 1 ml TE buffer (10 mM Tris, 1 mM EDTA, pH: 8.0) to regulate the density to a 4 McFarland standard. Then, they were heated at 100°C for 10 min. The suspension was centrifuged at 2500 rpm for 10 minutes and the supernatant was used for AP-PCR. DNA in the supernatant was quantitated by spectrophotometry at an optical density of 260 nm. PCR mixtures were prepared in 100 μl of 1X buffer (10 mM Tris-HCl, 50 mM KCl, 2.5 mM MgCl_2_) and contained 1 μg DNA, 0.1 mM each dNTP, 2.5 U *Taq *polymerase and 30 pmol of pBR322 *Sal*I primer (AGTCATGCCCCGCGC). PCR was initiated with five cycles of low stringency, which included a denaturing step at 95°C for 1 min, annealing of the primer at 28°C for 1 min, and 2 min of extension at 72°C. After the initial 5 cycles, 55 additional cycles were conducted with annealing of the primer at 50°C. The reaction was terminated with a final extension cycle at 72°C for 10 min.

Samples were electrophoresed in a 1.5% agarose gel (Sigma, Germany) in 1X Tris-borate-EDTA buffer for 90 min at 100 V and visualized under UV light after staining with ethidium bromide. To ensure reproducibility, all amplifications were done in duplicate and were also repeated using DNA extracted on different days. Dice coefficients of similarity were calculated for every pair of isolates by visual comparison of restriction patterns. If DNA profiles of isolates were indistinguishable or differing by only three or fewer DNA band shifts, then, the isolates were deemed same or related and included in the same pattern [13].

### Statistical analysis

Chi-square test (Fisher's exact test when necessary) was used.

## Abreviations

SXT: Trimethoprim/sulfamethoxazole

CIP: Ciprofloxacin

MIC: Minimal inhibitory concentration

TLc: Ticarcillin/clavulanate

NCCLS: The National Committee for Clinical Laboratory Standards

AP-PCR: The arbitrarily primed PCR

CAZ: Ceftazidime

CPM: Cefepime

PIP: Piperacillin

PTZ: Piperacillin/tazobactam

ATCC: The American Type Culture Collection

## Authors' contributions

All the authors have read and approved the final manuscript and contributed equally to the manuscript. MTO drafted the manuscript, participated in the susceptibility test studies, searched the literature and reviews. SG, BO and BA collected bacteria and participated in the susceptibility test studies. SB carried out the AP-PCR studies. MTO and SG performed the statistical analysis and gave the final approval of the version to be published.

## Figures and Tables

**Table 1:** Susceptibility of *S. maltophilia *obtained by the three methods studied (N = 52)

	Percentage susceptibility*			Agar MIC (mg/L)		
	
Agent	Disc diffusion	E Test	Agar dilution (MIC breakpoint)	MIC_50_	MIC_90_	MIC Range
Ceftazidime	67.3	63.5	50.0 (≤ 8 mg/L)	8	256	0.25->256
Cefepime	30.8	42.3	34.6 (≤ 8 mg/L)	16	64	1–64
Piperacillin	34.6	15.4	26.9 (≤ 16 mg/L)	64	256	1->256
PTZ	61.5	46.2	42.3 (≤ 16/4 mg/L)	32/4	128/4	4/4->256/4
TLc	84.6	90.4	73.1 (≤ 16/2 mg/L)	8/2	64/2	0.5/2->256/2
Ciprofloxacin	92.3	92.3	53.8 (≤ 1 mg/L)	1	4	0.5–16
SXT	98.1	98.1	98.1 (≤ 2/38 mg/L)	0.5/9.5	1/19	0.125/2.375- 128/2432

PTZ: Piperacillin/tazobactam, TLc: Ticarcillin/clavulanate, SXT: Trimethoprim/sulfamethoxazol

**Table 2:** Correlation of susceptibility test methods for 52 *S. maltophilia *strains

	% Discrepance							
		
	Very Major		Major Error		Minor Error		% Correlation	
		
N = 52	Agar-disc	Agar-E-test	Agar-disc	Agar-E-test	Agar-disc	Agar-E-test	Agar-disc	Agar-E-test
Ceftazidime	5.8	5.8	0	0	17.3	13.4	76.9	80.8
Cefepime	0	1.9	0	0	11.5	23.1	88.5	75.0
Piperacillin	0	0	1.9	1.9	38.5	38.5	59.6	59.6
PTZ	1.9	0	0	1.9	34.6	23.1	63.5	75.0
TLc	0	1.9	1.9	0	17.3	19.2	80.8	78.9
Ciprofloxacin	5.8	5.8	0	0	32.7	32.7	61.5	61.5
SXT	0	0	0	0	0	0	100	100

PTZ: Piperacillin/tazobactam, TLc: Ticarcillin/clavulanate, SXT: Trimethoprim/sulfamethoxazole
